# 4,11-Diaza-1,8-diazo­niacyclo­tetra­decane bis­(pyridine-2-carboxyl­ate) dihydrate

**DOI:** 10.1107/S1600536809037258

**Published:** 2009-09-19

**Authors:** Nam-Ho Kim, Kwang Ha

**Affiliations:** a School of Applied Chemical Engineering, The Research Institute of Catalysis, Chonnam National University, Gwangju 500-757, Republic of Korea

## Abstract

The asymmetric unit of the title compound, C_10_H_26_N_4_^2+^·2C_6_H_4_NO_2_^−^·2H_2_O, consists of half of a doubly protonated 1,4,8,11-tetra­azacyclo­tetra­decane (cyclam) dication, a pyridine-2-carboxyl­ate anion and a solvent water mol­ecule. The complete dication is generated by a crystallographic centre and adopts an endodentate conformation which may be influenced by intra­molecular N—H⋯N hydrogen bonding. The carboxyl­ate group of the anion appears to be delocalized on the basis of the C—O bond lengths [1.257 (2) and 1.250 (2) Å]. In the crystal structure, the components are linked by inter­molecular N—H⋯O, N—H⋯N and O—H⋯O hydrogen bonds.

## Related literature

For the crystal structures of [H_2_(cyclam)]*X* [*X* = (ClO_4_)_2_ or Cl_2_], see: Nave & Truter (1974 ▶); Kim *et al.* (2009 ▶). For the crystal structures of [H_4_(cyclam)]*X·n*H_2_O [*X* = Cl_4_, Br_4_, (ClO_4_)_4_, (SCN)_4_, (SO_4_)_2_ or (*p*-CH_3_C_6_H_4_SO_3_)_4_], see: Robinson *et al.* (1989 ▶); Subramanian & Zaworotko (1995 ▶). For the structure of pyridine-2-carboxylic acid, see: Hamazaki *et al.* (1998 ▶). For the crystal structures of pyridine-2-carboxyl­ate compounds, see: Kim & Ha (2009*a* ▶,*b* ▶).
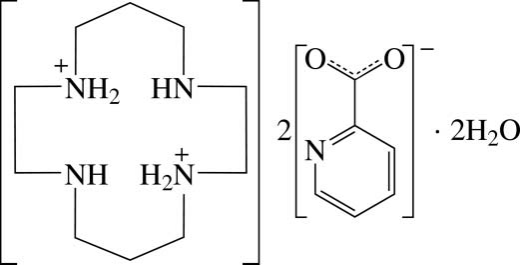


## Experimental

### 

#### Crystal data

C_10_H_26_N_4_^2+^·2C_6_H_4_NO_2_^−^·2H_2_O
*M* *_r_* = 482.58
Monoclinic, 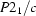

*a* = 10.2746 (8) Å
*b* = 12.0551 (9) Å
*c* = 10.3244 (8) Å
β = 93.104 (2)°
*V* = 1276.92 (17) Å^3^
*Z* = 2
Mo *K*α radiation
μ = 0.09 mm^−1^
*T* = 200 K
0.22 × 0.17 × 0.11 mm

#### Data collection

Bruker SMART 1000 CCD diffractometer
Absorption correction: multi-scan (*SADABS*: Sheldrick, 1996 ▶) *T*_min_ = 0.868, *T*_max_ = 1.000
9364 measured reflections
3152 independent reflections
1540 reflections with *I* > 2σ(*I*)
*R*_int_ = 0.070

#### Refinement

*R*[*F* ^2^ > 2σ(*F* ^2^)] = 0.055
*wR*(*F* ^2^) = 0.123
*S* = 1.02
3152 reflections
154 parameters
H-atom parameters constrained
Δρ_max_ = 0.22 e Å^−3^
Δρ_min_ = −0.25 e Å^−3^

### 

Data collection: *SMART* (Bruker, 2007 ▶); cell refinement: *SAINT* (Bruker, 2007 ▶); data reduction: *SAINT*; program(s) used to solve structure: *SHELXTL* (Sheldrick, 2008 ▶); program(s) used to refine structure: *SHELXTL*; molecular graphics: *ORTEP-3 for Windows* (Farrugia, 1997 ▶) and *PLATON* (Spek, 2009 ▶); software used to prepare material for publication: *SHELXTL*.

## Supplementary Material

Crystal structure: contains datablocks global, I. DOI: 10.1107/S1600536809037258/nk2004sup1.cif

Structure factors: contains datablocks I. DOI: 10.1107/S1600536809037258/nk2004Isup2.hkl

Additional supplementary materials:  crystallographic information; 3D view; checkCIF report

## Figures and Tables

**Table 1:** Hydrogen-bond geometry (Å, °)

*D*—H⋯*A*	*D*—H	H⋯*A*	*D*⋯*A*	*D*—H⋯*A*
N2—H21⋯O3	0.92	2.02	2.932 (2)	170
N3—H32⋯N2	0.92	2.53	2.926 (3)	106
N3—H32⋯N2^i^	0.92	2.08	2.846 (3)	139
N3—H31⋯O1^ii^	0.92	1.86	2.749 (2)	161
N3—H31⋯N1^ii^	0.92	2.46	3.039 (2)	121
O3—H3*A*⋯O1^iii^	0.84	1.99	2.808 (2)	165
O3—H3*B*⋯O2^iv^	0.84	1.91	2.739 (2)	168

Symmetry codes: (i) 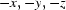; (ii) 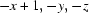; (iii) 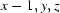; (iv) 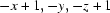.

**Fig. 1.**
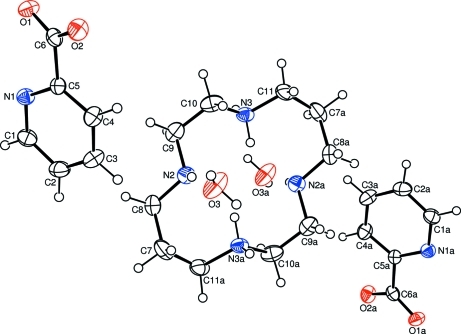
The structure of (I), with displacement ellipsoids drawn at the 40% probability level for non-H atoms [Symmetry code: (a) -x, -y, -z].

**Fig. 2.**
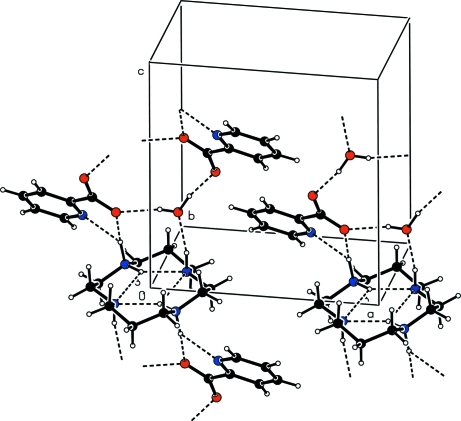
View of the unit-cell contents of (I). Hydrogen bonding interactions are drawn with dashed lines.

## supplementary crystallographic information

### Comment

The asymmetric unit of the title compound, C_10_H_26_N_4_^2+^.2C_6_H_4_NO_2_^-^.2H_2_O, (I), consists of half of a doubly protonated 1,4,8,11-tetraazacyclotetradecane (cyclam) dication, a pyridine-2-carboxylate anion and a solvent water molecule (Fig. 1). The macrocyclic dication contains two protonated N atoms and two secondary amine N atoms, and is located on a centre of inversion. The dication adopts an endodentate conformation with the N atoms oriented towards the centre of the macrocyclic cavity. The conformation may be stabilized by intramolecular N—H···N hydrogen bonding (Table 1 and Fig. 2). The N2—C9—C10—N3 torsion angle of 66.5 (2)° displays the *gauche* conformation for the group within the dication. A similar conformation is also observed in the structures cyclam (Robinson *et al.*, 1989) and [H_2_(cyclam)]*X* [*X* = (ClO_4_)_2_ or Cl_2_] (Nave & Truter, 1974; Kim *et al.*, 2009). Unlike cyclam and the dication, the tetracation, [H_4_(cyclam)]^4+^, adopts an exodentate conformation, in which all four N atoms are oriented away from the ring cavity, occupying positions as far away as possible from each other on the ring periphery (Robinson *et al.*, 1989; Subramanian & Zaworotko, 1995). The protonated N—C bond lengths (N3—C10/C11: 1.482 (3)/1.487 (3) Å) are longer than unprotonated N—C bond lengths (N2—C8/C9: 1.468 (3)/1.459 (3) Å). The carboxylate group of the anion appears to be delocalized on the basis of the C—O bond lengths (C—O: 1.250 (2) and 1.257 (2) Å). The components of the crystal structure are linked by intermolecular N—H···O, N—H···N and O—H···O hydrogen bonds into one-dimensional chains along [001] (Table 1 and Fig. 2).

### Experimental

A solution of 1,4,8,11-tetraazacyclotetradecane (0.100 g, 0.50 mmol) and pyridine-2-carboxylic acid (0.123 g, 1.00 mmol) in H_2_O (10 ml) was stirred for 3 h at 60 °C. The solvent was removed under vacuum and the residue was washed with acetone/ether, to give a white powder (0.221 g). Crystals suitable for X-ray analysis were obtained by slow evaporation from an acetone solution.

### Refinement

H atoms were positioned geometrically and allowed to ride on their respective parent atoms [C—H = 0.95 (CH) or 0.99 (CH_2_) Å, N—H = 0.92 Å, O—H = 0.84 Å and *U*_iso_(H) = 1.2*U*_eq_(C, N) or 1.5*U*_eq_(O)].

### Crystal data

**Table:**

C_10_H_26_N_4_^2+^·2C_6_H_4_NO_2_^−^·2(H_2_O)	*F*(000) = 520
*M**_r_* = 482.58	*D*_x_ = 1.255 Mg m^−^^3^
Monoclinic, *P*2_1_/*c*	Mo *K*α radiation, λ = 0.71073 Å
Hall symbol: -P 2ybc	Cell parameters from 1456 reflections
*a* = 10.2746 (8) Å	θ = 2.6–24.3°
*b* = 12.0551 (9) Å	µ = 0.09 mm^−^^1^
*c* = 10.3244 (8) Å	*T* = 200 K
β = 93.104 (2)°	Block, colourless
*V* = 1276.92 (17) Å^3^	0.22 × 0.17 × 0.11 mm
*Z* = 2	

### Data collection

**Table:**

Bruker SMART 1000 CCD diffractometer	3152 independent reflections
Radiation source: fine-focus sealed tube	1540 reflections with *I* > 2σ(*I*)
graphite	*R*_int_ = 0.070
ω scans	θ_max_ = 28.3°, θ_min_ = 2.0°
Absorption correction: multi-scan (*SADABS*: Sheldrick, 1996)	*h* = −11→13
*T*_min_ = 0.868, *T*_max_ = 1.000	*k* = −16→15
9364 measured reflections	*l* = −13→13

### Refinement

**Table:**

Refinement on *F*^2^	Primary atom site location: structure-invariant direct methods
Least-squares matrix: full	Secondary atom site location: difference Fourier map
*R*[*F*^2^ > 2σ(*F*^2^)] = 0.055	Hydrogen site location: inferred from neighbouring sites
*wR*(*F*^2^) = 0.123	H-atom parameters constrained
*S* = 1.02	*w* = 1/[σ^2^(*F*_o_^2^) + (0.0315*P*)^2^] where *P* = (*F*_o_^2^ + 2*F*_c_^2^)/3
3152 reflections	(Δ/σ)_max_ < 0.001
154 parameters	Δρ_max_ = 0.22 e Å^−^^3^
0 restraints	Δρ_min_ = −0.25 e Å^−^^3^

### Special details

**Table:**

Geometry. All e.s.d.'s (except the e.s.d. in the dihedral angle between two l.s. planes) are estimated using the full covariance matrix. The cell e.s.d.'s are taken into account individually in the estimation of e.s.d.'s in distances, angles and torsion angles; correlations between e.s.d.'s in cell parameters are only used when they are defined by crystal symmetry. An approximate (isotropic) treatment of cell e.s.d.'s is used for estimating e.s.d.'s involving l.s. planes.
Refinement. Refinement of *F*^2^ against ALL reflections. The weighted *R*-factor *wR* and goodness of fit *S* are based on *F*^2^, conventional *R*-factors *R* are based on *F*, with *F* set to zero for negative *F*^2^. The threshold expression of *F*^2^ > σ(*F*^2^) is used only for calculating *R*-factors(gt) *etc*. and is not relevant to the choice of reflections for refinement. *R*-factors based on *F*^2^ are statistically about twice as large as those based on *F*, and *R*- factors based on ALL data will be even larger.

### Fractional atomic coordinates and isotropic or equivalent isotropic displacement parameters (Å^2^)

**Table:**

	*x*	*y*	*z*	*U*_iso_*/*U*_eq_	
O1	0.84295 (15)	0.05500 (12)	0.30790 (13)	0.0377 (4)	
O2	0.71238 (16)	0.00216 (13)	0.46269 (15)	0.0433 (5)	
N1	0.67291 (18)	0.21986 (15)	0.23751 (17)	0.0333 (5)	
C1	0.5839 (2)	0.29517 (19)	0.1972 (2)	0.0383 (6)	
H1	0.6109	0.3538	0.1434	0.046*	
C2	0.4558 (2)	0.2926 (2)	0.2290 (2)	0.0434 (6)	
H2	0.3960	0.3481	0.1984	0.052*	
C3	0.4166 (2)	0.2074 (2)	0.3066 (2)	0.0449 (7)	
H3	0.3282	0.2017	0.3283	0.054*	
C4	0.5066 (2)	0.1306 (2)	0.3524 (2)	0.0384 (6)	
H4	0.4818	0.0725	0.4082	0.046*	
C5	0.6339 (2)	0.13915 (18)	0.31597 (19)	0.0278 (5)	
C6	0.7385 (2)	0.05841 (18)	0.3658 (2)	0.0312 (5)	
N2	0.14631 (18)	0.09306 (16)	0.05713 (17)	0.0374 (5)	
H21	0.1475	0.0811	0.1452	0.045*	
N3	0.12754 (18)	−0.12813 (15)	−0.05896 (17)	0.0360 (5)	
H31	0.1456	−0.1194	−0.1447	0.043*	
H32	0.0565	−0.0845	−0.0438	0.043*	
C7	0.0412 (3)	0.2719 (2)	0.0986 (3)	0.0510 (7)	
H7A	0.0561	0.3529	0.0946	0.061*	
H7B	0.0435	0.2503	0.1912	0.061*	
C8	0.1501 (2)	0.2132 (2)	0.0340 (2)	0.0446 (7)	
H8A	0.1424	0.2276	−0.0605	0.054*	
H8B	0.2349	0.2430	0.0681	0.054*	
C9	0.2570 (2)	0.0350 (2)	0.0057 (2)	0.0459 (7)	
H9A	0.3388	0.0598	0.0519	0.055*	
H9B	0.2625	0.0525	−0.0875	0.055*	
C10	0.2406 (2)	−0.0876 (2)	0.0229 (2)	0.0485 (7)	
H10A	0.3206	−0.1264	−0.0015	0.058*	
H10B	0.2270	−0.1042	0.1151	0.058*	
C11	0.0922 (3)	−0.24601 (19)	−0.0372 (3)	0.0487 (7)	
H11A	0.0928	−0.2609	0.0571	0.058*	
H11B	0.1577	−0.2949	−0.0748	0.058*	
O3	0.11622 (16)	0.04470 (16)	0.33220 (14)	0.0609 (6)	
H3A	0.0351	0.0418	0.3385	0.091*	
H3B	0.1593	0.0314	0.4019	0.091*	

### Atomic displacement parameters (Å^2^)

**Table:**

	*U*^11^	*U*^22^	*U*^33^	*U*^12^	*U*^13^	*U*^23^
O1	0.0313 (10)	0.0450 (11)	0.0374 (9)	0.0095 (8)	0.0064 (8)	0.0074 (8)
O2	0.0425 (11)	0.0484 (11)	0.0389 (9)	−0.0014 (8)	0.0010 (8)	0.0163 (8)
N1	0.0335 (12)	0.0338 (11)	0.0327 (10)	0.0028 (9)	0.0026 (9)	0.0033 (9)
C1	0.0439 (16)	0.0346 (14)	0.0361 (13)	0.0048 (12)	−0.0003 (12)	0.0055 (11)
C2	0.0398 (16)	0.0423 (16)	0.0476 (15)	0.0118 (13)	−0.0042 (13)	−0.0027 (13)
C3	0.0305 (15)	0.0573 (18)	0.0473 (15)	0.0062 (13)	0.0050 (12)	0.0018 (14)
C4	0.0377 (15)	0.0464 (15)	0.0314 (13)	−0.0027 (13)	0.0038 (11)	0.0052 (12)
C5	0.0289 (13)	0.0303 (13)	0.0239 (11)	−0.0024 (11)	−0.0012 (10)	−0.0007 (10)
C6	0.0365 (15)	0.0284 (13)	0.0281 (12)	−0.0041 (11)	−0.0045 (11)	−0.0015 (11)
N2	0.0427 (13)	0.0406 (12)	0.0296 (10)	0.0016 (10)	0.0071 (9)	−0.0003 (9)
N3	0.0392 (12)	0.0361 (12)	0.0333 (10)	0.0082 (10)	0.0086 (9)	0.0035 (9)
C7	0.074 (2)	0.0301 (14)	0.0499 (16)	−0.0049 (14)	0.0144 (16)	0.0006 (12)
C8	0.0459 (17)	0.0431 (16)	0.0448 (15)	−0.0104 (13)	0.0036 (13)	0.0033 (13)
C9	0.0392 (16)	0.0610 (18)	0.0377 (14)	−0.0040 (14)	0.0035 (12)	−0.0116 (13)
C10	0.0424 (16)	0.0643 (19)	0.0383 (14)	0.0160 (14)	−0.0020 (13)	−0.0044 (13)
C11	0.0565 (18)	0.0364 (16)	0.0554 (17)	0.0108 (13)	0.0231 (15)	0.0159 (13)
O3	0.0392 (11)	0.1048 (16)	0.0385 (10)	−0.0009 (11)	0.0012 (9)	0.0222 (10)

### Geometric parameters (Å, °)

**Table:**

O1—C6	1.257 (2)	N3—H31	0.9200
O2—C6	1.250 (2)	N3—H32	0.9200
N1—C1	1.339 (3)	C7—C8	1.509 (3)
N1—C5	1.341 (2)	C7—C11^i^	1.511 (4)
C1—C2	1.374 (3)	C7—H7A	0.9900
C1—H1	0.9500	C7—H7B	0.9900
C2—C3	1.377 (3)	C8—H8A	0.9900
C2—H2	0.9500	C8—H8B	0.9900
C3—C4	1.374 (3)	C9—C10	1.499 (3)
C3—H3	0.9500	C9—H9A	0.9900
C4—C5	1.385 (3)	C9—H9B	0.9900
C4—H4	0.9500	C10—H10A	0.9900
C5—C6	1.519 (3)	C10—H10B	0.9900
N2—C9	1.459 (3)	C11—C7^i^	1.511 (4)
N2—C8	1.468 (3)	C11—H11A	0.9900
N2—H21	0.9200	C11—H11B	0.9900
N3—C10	1.482 (3)	O3—H3A	0.8400
N3—C11	1.487 (3)	O3—H3B	0.8400
			
C1—N1—C5	117.3 (2)	C11^i^—C7—H7A	108.9
N1—C1—C2	123.9 (2)	C8—C7—H7B	108.9
N1—C1—H1	118.1	C11^i^—C7—H7B	108.9
C2—C1—H1	118.1	H7A—C7—H7B	107.7
C1—C2—C3	118.0 (2)	N2—C8—C7	111.34 (19)
C1—C2—H2	121.0	N2—C8—H8A	109.4
C3—C2—H2	121.0	C7—C8—H8A	109.4
C4—C3—C2	119.4 (2)	N2—C8—H8B	109.4
C4—C3—H3	120.3	C7—C8—H8B	109.4
C2—C3—H3	120.3	H8A—C8—H8B	108.0
C3—C4—C5	119.0 (2)	N2—C9—C10	109.6 (2)
C3—C4—H4	120.5	N2—C9—H9A	109.8
C5—C4—H4	120.5	C10—C9—H9A	109.8
N1—C5—C4	122.4 (2)	N2—C9—H9B	109.8
N1—C5—C6	116.15 (19)	C10—C9—H9B	109.8
C4—C5—C6	121.4 (2)	H9A—C9—H9B	108.2
O2—C6—O1	126.1 (2)	N3—C10—C9	110.3 (2)
O2—C6—C5	116.1 (2)	N3—C10—H10A	109.6
O1—C6—C5	117.80 (19)	C9—C10—H10A	109.6
C9—N2—C8	112.69 (19)	N3—C10—H10B	109.6
C9—N2—H21	108.4	C9—C10—H10B	109.6
C8—N2—H21	108.5	H10A—C10—H10B	108.1
C10—N3—C11	114.79 (19)	N3—C11—C7^i^	110.9 (2)
C10—N3—H31	108.6	N3—C11—H11A	109.5
C11—N3—H31	108.6	C7^i^—C11—H11A	109.5
C10—N3—H32	108.6	N3—C11—H11B	109.5
C11—N3—H32	108.6	C7^i^—C11—H11B	109.5
H31—N3—H32	107.5	H11A—C11—H11B	108.0
C8—C7—C11^i^	113.3 (2)	H3A—O3—H3B	113.9
C8—C7—H7A	108.9		
			
C5—N1—C1—C2	−1.6 (3)	C4—C5—C6—O2	−16.4 (3)
N1—C1—C2—C3	−0.2 (4)	N1—C5—C6—O1	−17.0 (3)
C1—C2—C3—C4	2.0 (4)	C4—C5—C6—O1	164.4 (2)
C2—C3—C4—C5	−1.9 (3)	C9—N2—C8—C7	−174.9 (2)
C1—N1—C5—C4	1.7 (3)	C11^i^—C7—C8—N2	−68.7 (3)
C1—N1—C5—C6	−176.94 (19)	C8—N2—C9—C10	−175.26 (19)
C3—C4—C5—N1	0.0 (3)	C11—N3—C10—C9	−172.71 (19)
C3—C4—C5—C6	178.6 (2)	N2—C9—C10—N3	66.5 (2)
N1—C5—C6—O2	162.19 (18)	C10—N3—C11—C7^i^	165.97 (19)

Symmetry codes: (i) −*x*, −*y*, −*z*.

### Hydrogen-bond geometry (Å, °)

**Table:**

*D*—H···*A*	*D*—H	H···*A*	*D*···*A*	*D*—H···*A*
N2—H21···O3	0.92	2.02	2.932 (2)	170
N3—H32···N2	0.92	2.53	2.926 (3)	106
N3—H32···N2^i^	0.92	2.08	2.846 (3)	139
N3—H31···O1^ii^	0.92	1.86	2.749 (2)	161
N3—H31···N1^ii^	0.92	2.46	3.039 (2)	121
O3—H3A···O1^iii^	0.84	1.99	2.808 (2)	165
O3—H3B···O2^iv^	0.84	1.91	2.739 (2)	168

Symmetry codes: (i) −*x*, −*y*, −*z*; (ii) −*x*+1, −*y*, −*z*; (iii) *x*−1, *y*, *z*; (iv) −*x*+1, −*y*, −*z*+1.
